# Complete Genome Sequence of a Novel Hypovirus from the Phytopathogenic Fungus *Fusarium langsethiae*

**DOI:** 10.1128/genomeA.01722-16

**Published:** 2017-03-02

**Authors:** Pengfei Li, Xiaoguang Chen, Hao He, Dewen Qiu, Lihua Guo

**Affiliations:** State Key Laboratory for Biology of Plant Diseases and Insect Pests, Institute of Plant Protection, Chinese Academy of Agricultural Sciences, Beijing, China

## Abstract

We describe a novel positive single-stranded RNA virus, termed *Fusarium langsethiae* hypovirus 1 (FlHV1), from the isolate AH32 of the phytopathogenic fungus *Fusarium langsethiae*. The properties of FlHV1 permit assignment to the genus *Alphahypovirus* in the family *Hypoviridae*. This is the first report of a mycovirus identified in *F. langsethiae*.

## GENOME ANNOUNCEMENT

Mycoviruses (fungal viruses) are ubiquitous among all major taxonomic groups of fungi. Mycoviruses of the family *Hypoviridae*, which do not produce true virions or encode capsid proteins, have monopartite ssRNA genomes ranging from 9 to 13 kb in size (1, 2). Twelve reported virus species within the family *Hypoviridae* were isolated from six plant pathogenic fungi: *Cryphonectria parasitica*, *Sclerotinia sclerotiorum*, *Fusarium graminearum*, *F. poae*, *Valsa ceratosperma*, and *Phomopsis longicolla* (3, 4). Li et al. (3) proposed that the family *Hypoviridae* should contain two genera: *Alphahypovirus* and *Betahypovirus*, instead of three genera (3, 5, 6).

The AH32 strain of *Fusarium langsethiae* was cultured on potato dextrose agar plates overlaid with cellophane membranes for 4 days at 25°C in the dark. The mycelium mass was used for dsRNA extraction using the CF-11 cellulose chromatography method (with 16% ethanol concentration). The purified dsRNA and random primers (5′ GACGTCCAGATCGCGAATTCNNNNNN 3′) were used to synthesize cDNAs (TransGen). The resulting cDNAs were amplified using a single specific primer (5′ GACGTCCAGATCGCGAATTC 3′) and the 2×TransTaq High Fidelity (HiFi) PCR SuperMix (TransGen). The amplified PCR products were purified using an EasyPure Quick Gel extraction kit (TransGen), ligated to the PMD18-T vector and transformed into Trans5α chemically competent cells (TaKaRa) for sequencing. Based on the sequences obtained, dsRNA-specific primers were designed and used for RT-PCR. To clone the termini of the dsRNAs, we performed the 3′ RNA ligase-mediated rapid amplification of cDNA ends (RLM-RACE) protocol as described by Xie et al. (7) and Chiba et al. (8).

The genome of *Fusarium langsethiae* hypovirus 1 (FlHV1) is 12,839 nucleotides (nt) long, excluding the poly (A) tail. The genome contains a single large putative open reading frame (ORF) flanked by two untranslated regions (UTRs) at the 5′ and 3′ termini. The ORF, beginning at AUG (nt positions 476 to 478) and terminating at UAA (nt positions 12329 to 12331), was predicted to encode a polyprotein of 3,951 amino acid (aa) residues, with a calculated molecular weight of 447.1 kDa. The deduced polyprotein contains three conserved domains of papain-like protease (Pro), RNA-dependent RNA polymerase (RdRp), and viral RNA helicase (Hel), which are contained in all of the members of the *Hypoviridae* family. The polyprotein shared the highest aa identities with *Fusarium poae* hypovirus 1 (FpHV1) (79%; BAV56305) and *Fusarium graminearum* hypovirus 2 (FgHV2) (77.7%; AKB94065). Multiple alignments and phylogenetic analyses of the viral polyproteins and the conserved domain (RdRp and Hel) aa sequences using the neighbor-joining method revealed that FlHV1 formed a well-supported phylogenetic branch together with FgHV2 and FpHV1, and was grouped in a big clade, *Alphahypovirus*, including *Cryphonectria* hypovirus 1 (CHV1), CHV2, FgHV1, FgHV2, FpHV1, and *Sclerotinia sclerotiorum* hypovirus 2 (SsHV2). Although Hu et al. and Khalifa and Pearson have suggested that a third distinct genus, *Gammahypovirus* should be located in the family *Hypoviridae* (5, 6), the isolation of an increasing number of novel hypoviruses will support the suggestion that the members of *Hypoviridae* are divided into two major groups: *Alphahypovirus* and *Betahypovirus*, as Li et al. (3) and Yaegashi et al. (9) have described.

### Accession number(s).

The full-length viral genomic sequence of FlHV1 from *Fusarium langsethiae* strain AH32 was deposited in GenBank under the accession number KY120321.

## References

[1] Ghabrial SA, Castón JR, Jiang D, Nibert ML, Suzuki N 2015 50-plus years of fungal viruses. Virology 479–480:356–368. doi:10.1016/j.virol.2015.02.034. 25771805

[2] Xie J, Jiang D 2014 New insights into mycoviruses and exploration for the biological control of crop fungal diseases. Annu Rev Phytopathol 52:45–68. doi:10.1146/annurev-phyto-102313-050222. 25001452

[3] Li P, Zhang H, Chen X, Qiu D, Guo L 2015 Molecular characterization of a novel hypovirus from the plant pathogenic fungus *Fusarium graminearum*. Virology 481:151–160. doi:10.1016/j.virol.2015.02.047. 25781585

[4] Osaki H, Sasaki A, Nomiyama K, Tomioka K 2016 Multiple virus infection in a single strain of *Fusarium poae* shown by deep sequencing. Virus Genes 52:835–847. doi:10.1007/s11262-016-1379-x. 27550368

[5] Hu Z, Wu S, Cheng J, Fu Y, Jiang D, Xie J 2014 Molecular characterization of two positive-strand RNA viruses co-infecting a hypovirulent strain of *Sclerotinia sclerotiorum*. Virology 464–465:450–459. doi:10.1016/j.virol.2014.07.007. 25104554

[6] Khalifa ME, Pearson MN 2014 Characterisation of a novel hypovirus from *Sclerotinia sclerotiorum* potentially representing a new genus within the *Hypoviridae*. Virology 464–465:441–449. doi:10.1016/j.virol.2014.07.005. 25108682

[7] Xie J, Wei D, Jiang D, Fu Y, Li G, Ghabrial SA, Peng Y 2006 Characterization of debilitation-associated mycovirus infecting the plant-pathogenic fungus *Sclerotinia sclerotiorum*. J Gen Virol 87:241–249. doi:10.1099/vir.0.81522-0. 16361437

[8] Chiba S, Salaipeth L, Lin YH, Sasaki A, Kanematsu S, Suzuki N 2009 A novel bipartite double-stranded RNA mycovirus from the white root rot fungus *Rosellinia necatrix*: molecular and biological characterization, taxonomic considerations, and potential for biological control. J Virol 83:12801–12812. doi:10.1128/JVI.01830-09. 19828620 PMC2786845

[9] Yaegashi H, Kanematsu S, Ito T 2012 Molecular characterization of a new hypovirus infecting a phytopathogenic fungus, *Valsa ceratosperma*. Virus Res 165:143–150. doi:10.1016/j.virusres.2012.02.008. 22366520

